# Antioxidant Potential of Ethiopian Medicinal Plants and Their Phytochemicals: A Review of Pharmacological Evaluation

**DOI:** 10.1155/2023/1901529

**Published:** 2023-10-12

**Authors:** Gashaw Nigussie, Abolghasem Siyadatpanah, Roghayeh Norouzi, Eyob Debebe, Mekdelawit Alemayehu, Aman Dekebo

**Affiliations:** ^1^Armauer Hansen Research Institute, P.O. Box: 1005, Addis Ababa, Ethiopia; ^2^Department of Applied Chemistry, Adama Science and Technology University, P.O. Box 1888, Adama, Ethiopia; ^3^Department of Medical Microbiology, Faculty of Medicine, Infectious Diseases Research Center, Gonabad University of Medical Sciences, Gonabad, Iran; ^4^Department of Pathobiology, Faculty of Veterinary Medicine, University of Tabriz, Tabriz, Iran; ^5^Institute of Pharmaceutical Sciences, Adama Science and Technology University, P.O. Box 1888, Adama, Ethiopia

## Abstract

**Background:**

Free radicals are very reactive molecules produced during oxidation events that in turn initiate a chain reaction resulting in cellular damage. Many degenerative diseases in humans, including cancer and central nervous system damage, are caused by free radicals. Scientific evidence indicates that active compounds from natural products can protect cells from free radical damage. As a result, the aim of this review is to provide evidence of the use of diverse Ethiopian medicinal plants with antioxidant properties that have been scientifically validated in order to draw attention and foster further investigations in this area.

**Methods:**

The keywords antioxidant, radical scavenging activities, reactive oxygen species, natural product, Ethiopian Medicinal plants, and 2, 2-Diphenyl-1-picrylhydrazyl radical scavenging assay (DPPH) were used to identify relevant data in the major electronic scientific databases, including Google Scholar, ScienceDirect, PubMed, Medline, and Science domain. All articles with descriptions that were accessed until November 2022 were included in the search strategy.

**Results:**

A total of 54 plant species from 33 families were identified, along with 46 compounds isolated. More scientific studies have been conducted on plant species from the Brassicaceae (19%), Asphodelaceae (12%), and Asteraceae (12%) families. The most used solvent and extraction method for plant samples are methanol (68%) and maceration (88%). The most examined plant parts were the leaves (42%). Plant extracts (56%) as well as isolated compounds (61%) exhibited significant antioxidant potential. The most effective plant extracts from Ethiopian flora were *Bersama abyssinica*, *Solanecio gigas*, *Echinops kebericho*, *Verbascum sinaiticum*, *Apium leptophyllum*, and *Crinum abyssinicum*. The best oxidative phytochemicals were Rutin (**7**), Flavan-3-ol-7-*O*-glucoside (**8**), Myricitrin (**13**), Myricetin-3-O-arabinopyranoside (**14**), 7-*O*-Methylaloeresin A (**15**), 3-Hydroxyisoagatholactone (**17**), *β*-Sitosterol-3-*O*-*β*-D-glucoside (**22**), Microdontin A/B (**24**), and Caffeic acid (**39**).

**Conclusion:**

Many crude extracts and compounds exhibited significant antioxidant activity, making them excellent candidates for the development of novel drugs. However, there is a paucity of research into the mechanisms of action as well as clinical evidence supporting some of these isolated compounds. To fully authenticate and then commercialize, further investigation and systematic analysis of these antioxidant-rich species are required.

## 1. Introduction

The generation of reactive oxygen species (ROS) and other free radicals during metabolism is a natural activity that is adequately compensated for by an elaborate endogenous antioxidant defense mechanism [1]. Oxidative stress results from the overproduction of free radicals and an imbalance in their elimination. In diseases including cancer, cardiovascular disease, inflammatory disease, and cataract development, oxidative damage at the cellular or subcellular level is now considered a major event. Reactive oxygen radicals exert an adverse effect on cells due to their ability to promote lipid peroxidation in cellular membranes, which results in lipid peroxides that severely damage membranes and cause chromosomal damage through membrane contact [2, 3]. Hydrogen peroxide, superoxide anion, and hydroxyl radicals are examples of oxygen free radicals that have been linked to the development of several pathological disorders, including diabetes, atherosclerosis, ischemia, and inflammatory diseases. In many cases, the first stage of these disorders is endothelial cell damage. These oxidants can be immediately scavenged by the antioxidant enzymes superoxide dismutase (SOD), catalase (CAT), and glutathione peroxidase (GPX), which are present intracellular or released into the extracellular milieu. They can also prevent these oxidants from becoming toxic species. It is well known that ROS and reactive metabolic intermediates produced by different chemical carcinogens play a significant role in cell damage as well as the beginning and development of carcinogenesis. In recent decades, there has been a growing understanding of the connection between nutrition and chronic diseases, particularly cancer and cardiovascular disorders. Many degenerative diseases, including cancer, cataract, type 2 diabetes, neurological diseases, cardiovascular diseases, and inflammatory diseases, as well as the natural aging process, are now thought to be primarily caused by oxidative stress. Consequently, there is currently a lot of interest in the potential role of natural antioxidants in delaying or suppressing oxidative stress [4, 5]. Exogenous antioxidants need to be consumed or taken as supplements to maintain the body's endogenous antioxidant system. It has been appreciated that both nutrient and non-nutrient-rich diet components have antioxidant capabilities and consequent potential benefits. There has been a growing interest in natural antioxidants found abundantly in plants [6, 7]. Since the dawn of human civilization, medicinal plants have been identified and customarily used throughout the world [8, 9].

Medicinal plants are a rich source of novel drugs that form the ingredients in traditional systems of medicine [10, 11]. Most developing countries rely on traditional medicinal plants for their healthcare. Therefore, it should come as no surprise that some of these plants contain chemical compounds that have therapeutic potential and could be utilized to treat serious diseases like malaria, cancer, and pathogenic microbes [12]. According to studies, more than 80% of Ethiopians use plant-based traditional medicine as their primary healthcare system. This high adoption rate can be largely ascribed to the fact that it draws on locally accessible wild plant resources [13, 14]. This is in part because the vast majority of rural residents cannot access modern medical services because of their high cost, lack of transportation, and scarcity of healthcare centers [15]. However, the limited number of medicinal plants has been the focus of the available reviews on the antioxidant potential of Ethiopian natural products [16]. In spite of this, there is a paucity of comprehensive ethnopharmacological research review on Ethiopian antioxidant medicinal herbs. This review examined the phytochemistry of the plants used in traditional Ethiopian medicine as well as numerous investigations that have been done to scientifically validate their antioxidant potential. This evaluation may pave the way for additional complementary studies as well as the development of some readily available and affordable antioxidant phytomedicines, in line with the objectives of the WHO's “Traditional Medicine Strategy” [17].

## 2. Methodology

This review was compiled from various databases, including Google Scholar, ScienceDirect, PubMed, Medline, and Science domain from September 2022 to November 2022, to identify natural products from Ethiopian flora and fauna with antioxidant potential. Each database search was done independently. Until November 2022, original studies about antioxidant plants that were published in peer-reviewed journals were included in the study databases. The keywords antioxidant, radical scavenging activities, antiaging principles, reactive oxygen species, free radicals, natural product, 2, 2-Diphenyl-1-picrylhydrazyl radical scavenging assay (DPPH), and reducing properties were used to identify relevant data. All valuable data previously published in English have been gathered. The reviewers found relevant articles and gathered the following information from them: plant species, plant family, parts of the plant used, extraction methods, extraction solvent, IC_50_ values, and isolated compounds.

### 2.1. Categorization of Antioxidant Activities

For evaluating the *in vitro* antioxidant potencies of natural compounds and extracts, many techniques have been developed. These techniques are based on two important chemical processes: electron transfer reactions and hydrogen atom reactions. Electron transfer reactions are used to measure the following parameters to determine the antioxidant potencies of extracts and compounds using hydrogen atom transfer mechanisms: ferric reducing antioxidant power (FRAP), diphenyl-2-picryl-hydroxyl radical scavenging assay (DPPH), Trolox equivalent antioxidant capacity (TEAC), hydroxyl radical scavenging assay, superoxide anion radical scavenging assay, and nitric oxide radical scavenging [18]. Despite the recent increase in interest in antioxidant studies, it has been difficult to evaluate research findings from various research groups due to a lack of standardized assays [19]. To increase the reliability of the antioxidant results, more than one protocol was used, and the antioxidant potencies of natural products reviewed in this study were classified into three groups based on previous studies: high or significant antioxidant capacity with IC_50_ < 50 *μ*g/mL (extract) or IC_50_ < 10 *μ*g/mL (compounds), moderate antioxidant capacity with 50 < IC_50_ < 100 *μ*g/mL (extract) or 10 < IC_50_ < 20 *μ*g/mL (compounds), and low antioxidant capacity with IC_50_ > 100 *μ*g/mL (extract) or IC_50_ > 20 *μ*g/mL (compounds) [16, 20]. All activity data were converted to IC_50_ values in *μ*g/mL.

## 3. Result and Discussion

### 3.1. Promising Antioxidant Medicinal Plants from the Ethiopian Flora

**Figure 1:**
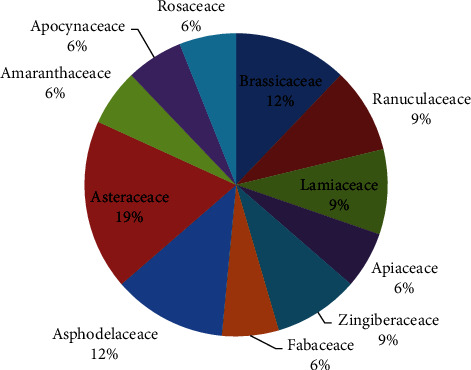
Percentage of the most well-investigated Ethiopian plant families for antioxidant activity.

The *in vitro* antioxidant activities of extracts from 54 plant species from 33 plant families were identified . Table 1 provides a summary of the plant species that were tested, their family, the portions of the plants that were utilized to generate the test samples, the solvent used during the extraction process, the assay methods, and their potencies based on the categorization/protocol used. This shows that Ethiopia has a diverse flora and that numerous people use several plant species for medicinal purposes [59]. Asteraceae 6 (19%), Brassicaceae 4 (12%), and Asphodelaceae 4 (12%) are the three plant families with the greatest antioxidant activity studied in Ethiopia (Figure 1 and Table 1).

**Figure 2:**
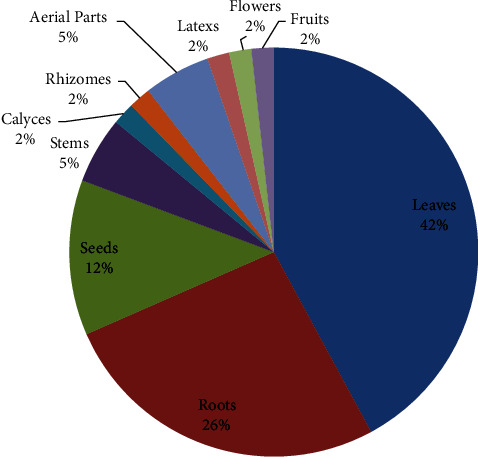
Plant parts investigated for their antioxidant potential.

The aforementioned family, which can be found in every floristic region of the country, may be the subject of this account [60]. Leaves 24 (42%) and roots 15 (26%) are the most investigated parts (Figure 2). This study indicates that using leaves for studies is crucial for medicinal plant conservation since, unlike with roots or whole plant collections, leaf harvesting may not be harmful to plants [61, 62].

Maceration (88%) is one of the most used plant sample extraction methods. Perhaps this is because solvent extraction, or more specifically, maceration, is one of the most popular and straightforward techniques for isolating plant antioxidants [63, 64]. Methanol is the most popular extraction solvent, although more polar solvents such as water and ethanol are frequently recommended in traditional preparations [65]. Surprisingly, in most studies, methanol (68%) plant extracts correlated with the antioxidant activity of the plant species studied. This is advantageous because it permits medicinal substances to absorb through the stomach lumen into the circulatory system, where they are required, following Lipinski's rules of 5 [66]. Therefore, active substances function through cell surface receptors, with polar components offering therapeutically significant potency *in vivo*. The antioxidant potential of plant extracts from 30 plants was significant (56%) (IC_50_ < 50 *μ*g/mL). The antioxidant activity of eight plant extracts was moderate (15%), with IC_50_ values ranging from 50 to 100 *μ*g/mL. With IC_50_ values greater than 100 *μ*g/mL, 14 plant extracts showed low (26%) antioxidant activities, whereas two plant extracts exhibited both significant and moderate (2%) antioxidant activities. This implies that Ethiopian medicinal herbs were found to have strong antioxidant properties, indicating that, if thoroughly examined, they might produce valuable pharmaceutical drugs for the treatment of oxidative stress disease.

### 3.2. Promising Antioxidant Phytochemicals Derived from the Ethiopian Flora

**Figure 3:**
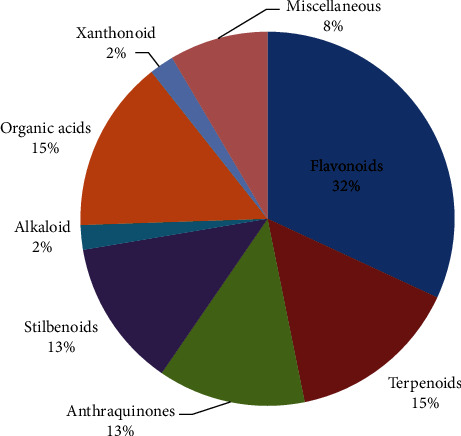
Percentage occurrence of antioxidant compounds isolated from Ethiopian medicinal plants.

More than 40 compounds from different chemical classes have so far been found in Ethiopian medicinal plants. Flavonoids 15 (32%), terpenoids 7 (15%), and organic acids 7 (15%) are the main components isolated from diverse plant species (Figure 3 and Table 2). Serial extraction, bioassay-guided extraction, successive fractionation using various polarity solvents, and column chromatography are the techniques used to isolate novel compounds for the plants of the species. The rising interest in using traditional medicine as an alternative and complementary therapy is encouraging activity-guided bioactive compound isolation to gain attention at the moment [70].

The significant (IC_50_ < 10 *μ*g/mL) antioxidant potential of 29 compounds was 61%. With IC_50_ values ranging from 10 to 20 *μ*g/mL, the antioxidant activity of 5 compounds was moderate (11%), and one compound exhibited both significant and moderate (3%) antioxidant activities, while 12 compounds with IC_50_ values higher than 20 *μ*g/mL exhibited low antioxidant activity (25%). The root of the plant species was frequently considered for investigation.

#### 3.2.1. Flavonoids

**Figure 4:**
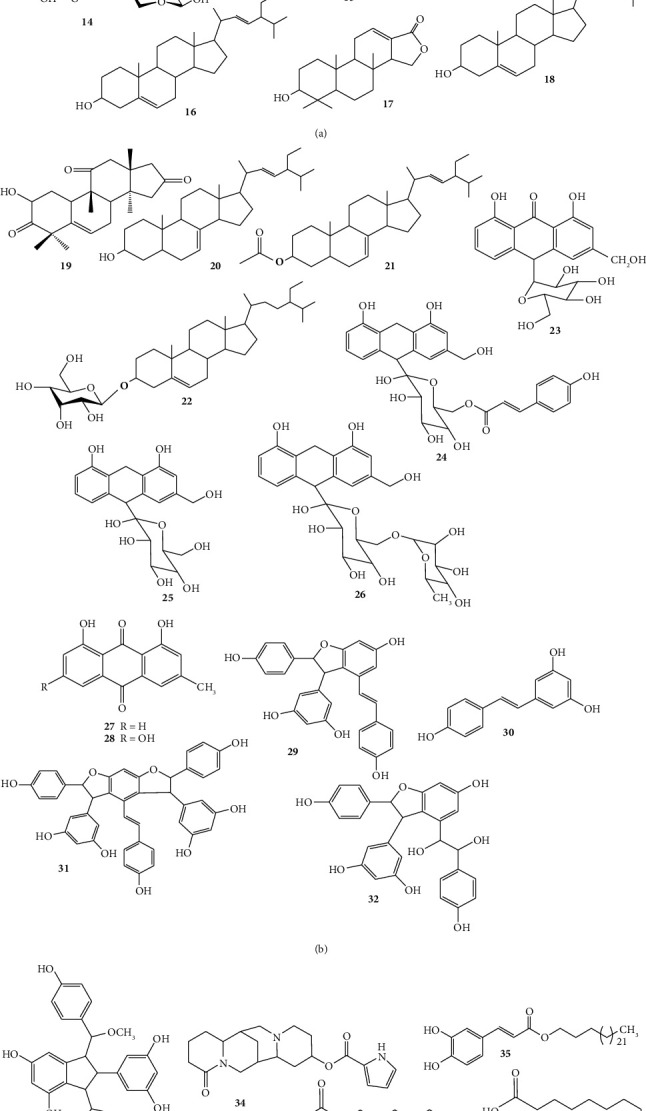
Antioxidant compounds isolated from Ethiopian flora.

**Figure 5:**
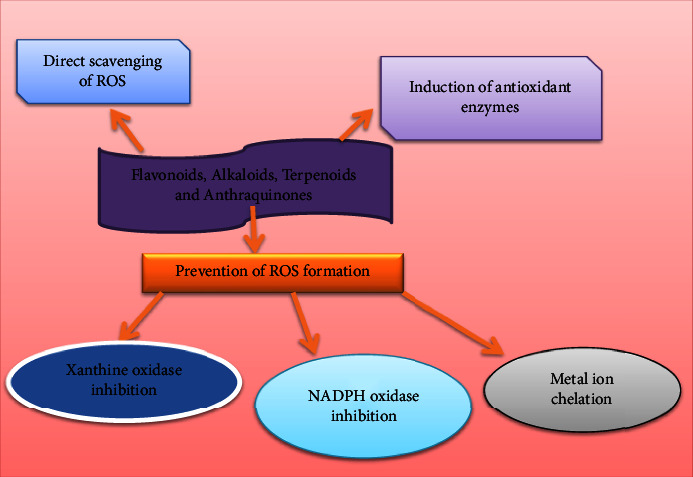
Mechanism of action of antioxidant effects of flavonoids, alkaloids, terpenoids, and anthraquinones. Flavonoids, alkaloids, terpenoids, and anthraquinones exert antioxidant effects by reactive oxygen species (ROS) scavenging, preventing ROS formation, and increasing production of antioxidant enzymes.

From ten plant species, 15 compounds (**1–15**) were isolated. Table 2 summarizes them, and Figure 4 depicts their chemical structures. The most effective compounds were Rutin (**7**) from *Cineraria abyssinica's* aqueous and methanol leaf extracts, Flavan-3-ol-7-O-glucoside (**8**) from *Hydnora johannis*' CH_2_Cl_2_/MeOH (1 : 1) root extracts, and 7-O-Methylaloeresin A (**15**) from *Aloe harlana's* leaf latex, with IC_50_ values of 3.53, 0.19, and 0.014 *μ*g/mL, respectively [29, 31, 68]. Flavonoids are the most abundant naturally occurring phenolic compounds well known for their antioxidant properties (Figure 5), which help in the prevention of a number of diseases including cancer, cardiovascular disease, and neurodegenerative diseases [71–74]. As a result, the presence of these significant compounds and the powerful antioxidant potential they exhibited indicate that, if rigorously screened, these compounds could provide medications of pharmaceutical relevance from those species.

#### 3.2.2. Terpenoids

Terpenoids represent the largest group of plant secondary metabolites [75]. There are tens of thousands of naturally occurring hydrocarbons, making them one of the classes of natural compounds with the most structural diversity. Terpenoids are categorized as hemiterpenes (C_5_), monoterpenes (C_10_), sesquiterpenes (C_15_), diterpenes (C_20_), triterpenes (C_30_), tetraterpenes or carotenoids (C_40_), and polyterpenes (C_*n*,*n*_ > 40) [75]. Numerous studies indicated that terpenoids and their derivatives exhibited antioxidant and antiaging properties (Figure 5), which help in the prevention of a number of diseases including cancer, cardiovascular disease, and neurodegenerative diseases [76–78]. Six plant species from Ethiopia's flora were studied for their antioxidant compounds. Seven compounds (**16–22**) were isolated, and **17**, **18**, **21,** and **22** of those compounds demonstrated significant antioxidant properties with IC_50_ values of 6.05, 2.72, 0.3, and 0.014 *μ*g/mL, respectively (Table 2 and Figure 4). The most effective compound (**22**), which is in line with the previous investigation, has been reported in the literature for its antioxidant activity [79–81].

#### 3.2.3. Anthraquinone

Anthraquinones, also known as anthracene diones or dioxoanthracenes, are significant quinones that make up a wide range of structurally different compounds of the polyketide family. It is essentially an organic compound that is aromatic. There are around 700 members of this group in fungi, lichens, and plants [82]. Many of them possess antimicrobial, antioxidant, anti-inflammatory, and antiviral properties [83, 84]. The mechanism of action of anthraquinones' antioxidant properties is demonstrated in Figure 5. In Table 2, the most promising recently discovered antioxidant anthraquinones derived from Ethiopian flora have been included. These include Aloin (**23**), Microdontin A/B (**24**), Aloin A/B (**25**), Aloinoside A/B (**26**), Chrysophanol (**27**), and Emodin (**28**), whose chemical structures are depicted in Figure 4. *Aloe harlana* (Asphodelaceae) [29], *Aloe schelpei* (Asphodelaceae) [30], and *Laggera tomentosa* (Asteraceae) [33] species were used to isolate the compounds. Compounds **24–26**, which had IC_50_ values of 0.07, 0.15, and 0.13 *μ*g/mL, were isolated from *Aloe schelpei* leaves' latex and showed significant antioxidant activity [30]. Compounds **27** and **28** were obtained by extracting the roots of *Laggera tomentosa* in methanol, and they demonstrated significant antioxidant activity, with IC_50_ values of 6.2 and 3.8 *μ*g/mL, respectively [33]. Compound **23** was derived from the leaves' latex of *Aloe harlana*, but it only has low antioxidant properties, with an IC_50_ value of 41.84 *μ*g/mL [29].

#### 3.2.4. Stilbenoids

Stilbenoids are a distinct class of phenolic compounds with C_6_-C_2_-C_6_ units as their basic structure [85]. Nowadays, natural stilbenoids are sold commercially as nutraceuticals [85]. According to a recent review, stilbenoids exhibited significant biological effects, including antioxidant, anti-inflammatory, cardioprotective, neuroprotective, antidiabetic, depigmentation, and cancer prevention and treatment [86–88]. Table 2 shows the most promising antioxidant stilbenoids from Ethiopian flora that have recently been published. Figure 4 illustrates the chemical structures of these compounds, which include *ε*-Viniferin (**29**), Trans-Resveratrol (**30**), Gnetin (**31**), *ε*-Viniferin Diol (**32**), and Parthenostilbenin (**33**). The compounds were isolated from the roots of *Cyphostemma cyphopetalum* (Vitaceae), and they demonstrated significant antioxidant activity with IC_50_ values ranging from 0.017 to 0.157 *μ*g/mL [69].

#### 3.2.5. Alkaloids

Alkaloids are secondary metabolites that were first described as pharmacologically active molecules largely made of nitrogen [89]. They are formed from lysine, tyrosine, and tryptophan, three of the few common amino acids. Plants have been shown to contain more than 12,000 alkaloids, representing more than 150 families, and about 20% of the “species of flowering plants” contain alkaloids [89]. The mechanism of action of alkaloids' antioxidant properties is demonstrated in Figure 5 [90]. Compound **33** was isolated from *Cadia purpurea* (Fabaceae), and it exhibits a low level of antioxidant activity, with an IC_50_ value of 58.44 *μ*g/mL [44].

#### 3.2.6. Organic Acid

Seven antioxidant organic acid compounds (**35–41**) that were isolated in the Ethiopian flora are listed in Table 2 along with a depiction of their chemical structure in Figure 4. Caffeic acid (**39**) and chlorogenic acid (**40**), two of such compounds, were isolated from the aerial parts of *Cheilanthes farinosa* (Pteridaceae), and they exhibited significant antioxidant activity with IC_50_ values of 4.19 and 8.01 g/mL, respectively [38].

#### 3.2.7. Xanthonoid

A xanthonoid is a chemical natural phenolic compound formed from the xanthone backbone [91]. Mangiferin is the best example, as it is a powerful therapeutic agent for treating a variety of diseases [92–94]. The antioxidant compound mangiferin (**42**), which was isolated from the leaves of *Bersama abyssinica*, had a significant antioxidant activity with an IC_50_ value of 6.72 *μ*g/mL [38].

#### 3.2.8. Miscellaneous Compounds

From three different plant species, four different compounds have been isolated (Table 2 and Figure 4). Di-(2-methylheptyl) phthalate (**43**) was isolated from the roots of *Cadia purpurea* (Fabaceae) [44], Ethyl (E)-octadec-8-enoate (**44**) and Penicilloitins B (**46**) were isolated from the roots of *Crinum abyssinicum* (Amaryllidaceae), and (4Z)-dodec-4-en-1-ol (**45**) was isolated from the roots of *Calotropis procera* (Apocynaceae) [24]. With an IC_50_ value of 3.3 *μ*g/mL, (4Z)-dodec-4-en-1-ol (**45**) exhibited the most significant antioxidant properties [24].

## 4. Conclusion and Future Prospects

Oxidative stress results from an excessive free radical formation that is out of balance with the elimination of those radicals. Oxidative stress has been linked to the etiology of cancer, inflammatory diseases, cardiovascular disease, and other serious diseases. Antioxidants are substances that impede oxidative processes, prolonging or suppressing oxidative stress in the process. Natural antioxidants that are present in plants are gaining popularity. From a safety perspective, herbs and spices are the most crucial objectives when looking for natural antioxidants. Strong antioxidant, anti-inflammatory, antimutagenic, and cancer-preventive properties are shared by a wide range of phenolic compounds found in spices that are frequently employed as food additives. The current review provides a summary of Ethiopian studies on potentially antioxidant-rich medicinal herbs. The article reviews draw attention to some active metabolites and plant extracts that have the potential to become brand-new drugs or improved plant medicines. A number of these natural products and secondary metabolites demonstrated and showed significant antioxidant properties. Based on the findings, the most effective oxidative plant extracts from Ethiopian flora were *Bersama abyssinica*, *Solanecio gigas*, *Echinops kebericho*, *Verbascum sinaiticum*, *Apium leptophyllum*, and *Crinum abyssinicum*. The best oxidative phytochemicals were rutin (**7**), flavan-3-ol-7-*O*-glucoside (**8**), myricitrin (**13**), myricetin-3-O-arabinopyranoside (**14**), 7-*O*-methylaloeresin A (**15**), 3-hydroxyisoagatholactone (**17**), beta-sitosterol (**18**), *β*-sitosterol-3-*O*-*β*-D-glucoside (**22**), microdontin A/B (**24**), aloin A/B (**25**), aloinoside A/B (**26**), chrysophanol (**27**), emodin (**28**), *ε*-viniferin (**29**), trans-resveratrol (**30**), gnetin H (**31**), *ε*-viniferin diol (**32**), parthenostilbenin B (**33**), and caffeic acid (**39**). It is hoped that competent researchers and interested individuals will investigate some of these plants and compounds further to provide a thorough verification and subsequently facilitate commercialization. The detailed isolation, characterization, mechanisms of action, safety investigations, quality control, and clinical trials on some of these herbs and their isolated compounds are far from satisfactory, although the majority of the studies examined are preliminary. Therefore, further *in vivo* studies on these species are needed, as well as a systematic analysis of these antioxidant-rich species.

## Figures and Tables

**Table 1:** Antioxidant potential of plant extracts from Ethiopian flora.

Plant	Family	Plant part investigated	Extraction method	Solvents	Assay methods	Inhibition/IC_50_	Antioxidant potential	Ref
*Hypoestes forskaolii*	Acanthaceae	Dried leaves	Maceration	Methanol	DPPH	15.7 *μ*g/mL	Significant	[21]
*Achyranthes aspera*	Amaranthaceae	Dried leaves	Maceration	Distilled water	DPPH	13510 *μ*g/mL	Low	[22]
*Amaranthus hybridus*	Amaranthaceae	Dried seeds	Maceration extraction	Methanol	DPPH	197.22 *μ*g/mL	Low	[23]
*Crinum abyssinicum*	Amaryllidaceae	Dried roots	Maceration extraction	DCM/methanol (1 : 1)	DPPH	4.1 *μ*g/mL	Significant	[24]
*Apium leptophyllum*	Apiaceae	Dried leaves	Hydrodistillation	Oil	DPPH	4.3 *μ*l/mL	Significant	[25]
*Trachyspermum ammi*	Apiaceae	Dried seeds	Maceration technique	Methanol	DPPH	74.4 *μ*g/mL	Moderate	[26]
*Calotropis procera*	Apocynaceae	Dried roots	Maceration extraction	Methanol	DPPH	4.3 *μ*g/mL	Significant	[24]
*Gomphocarpus fruticosus*	Apocynaceae	Dried leaves	Maceration extraction	Distilled water	DPPH	1640 *μ*g/mL	Low	[22]
*Dracaena angustifolia*	Asparagaceae	Dried leaves	Maceration extraction	Methanol	DPPH	25.59 *μ*g/mL	Significant	[27]
*Aloe debrana*	Asphodelaceae	Dried roots	Simultaneous distillation extraction	Distilled water and CH_2_Cl_2_	DPPH, H_2_O_2_	48.65 and 51.97 *μ*g/mL respectively	Significant, moderate	[28]
*Aloe harlana*	Asphodelaceae	Latex	—	—	DPPH	14.21 *μ*g/mL	Significant	[29]
*Aloe pulcherrima*	Asphodelaceae	Dried leaves	Maceration extraction	Distilled water	DPPH	420 *μ*g/mL	Low	[22]
*Aloe schelpei*	Asphodelaceae	Leaves' latex	—	—	DPPH	25.3 *μ*g/mL	Significant	[30]
*Cineraria abyssinica*	Asteraceae	Dried leaves	Maceration	Aqueous and methanol	DPPH	6.73 and 5.78 *μ*g/mL	Significant	[31]
*Echinops kebericho*	Asteraceae	Dried roots	Maceration extraction	Methanol crude extract and acetone fraction	DPPH	5.89 and 4.11 *μ*g/mL respectively	Significant	[32]
*Haplocarpha rueppelii*	Asteraceae	Dried leaves	Maceration extraction	Methanol	DPPH	35.2 *μ*g/mL	Significant	[23]
*Haplocarpha schimperi*	Asteraceae	Dried leaves	Maceration extraction	Methanol	DPPH	64.52 *μ*g/mL	Moderate	[23]
*Laggera tomentosa*	Asteraceae	Dried roots	Maceration extraction	EtOAc, and MeOH	DPPH	9.4 and 29 *μ*g/mL respectively	Significant	[33]
*Solanecio gigas*	Asteraceae	Dried stem bark	Maceration extraction	Methanol	DPPH	4.2 *μ*g/mL	Significant	[34]
*Brassica carinata*	Brassicaceae	Dried seeds	Maceration	Methanol	DPPH	5.85 mg/mL	Significant	[35]
*Eruca sativa*	Brassicaceae	Dried leaves	Maceration technique	Methanol	DPPH	150 *μ*g/mL	Low	[36]
*Erucastrum abyssinicum*	Brassicaceae	Dried leaves	Maceration extraction	Methanol	DPPH	100.58 *μ*g/mL	Low	[23]
*Raphanus sativus*	Brassicaceae	Dried leaves, roots	Maceration technique	Methanol	DPPH	160 and 450 *μ*g/mL respectively	Low	[36]
*Cucumis prophetarum*	Cucurbitaceae	Dried roots	Maceration extraction	Methanol	DPPH	28.9 *μ*g/mL	Significant	[37]
*Euclea racemosa*	Ebenaceae	Dried leaves	Soxhlet	Acetone	DPPH	11.3 *μ*g/mL	Significant	[38]
*Croton macrostachyus*	Euphorbiaceae	Dried root barks	Maceration	Ethanol	DPPH	128.6 *μ*g/mL	Low	[39]
*Albizia lebbeck*	Fabaceae	Dried stem bark	Maceration extraction	Methanol	DPPH	156 *μ*g/mL	Low	[40]
*Rhynchosia ferruginea*	Fabaceae	Dried roots	Maceration extraction	CH_2_Cl_2_/CH_3_OH	DPPH	17.7 *μ*g/mL	Significant	[41]
*Bersama abyssinica*	Francoaceae	Dried leaves	Maceration extraction, Soxhlet	Methanol	DPPH	5.35 and 7.5 *μ*g/mL	Significant	[38, 42]
*Salvia officinalis*	Lamiaceae	Dried aerial parts	Hydrodistillation	Oil	DPPH	4.65 *μ*g/mL	Significant	[43]
*Satureja punctata*	Lamiaceae	Dried aerial parts	Maceration extraction	Distilled water	DPPH	10 *μ*g/mL	Significant	[22]
*Thymus schimperi*	Lamiaceae	Dried leaves	Maceration technique	Methanol	DPPH	60.1 *μ*g/mL	Moderate	[26]
*Cadia purpurea*	Leguminosae	Dried roots	Maceration extraction	Ethanol	DPPH	12.9 *μ*g/mL	Significant	[44]
*Termitomyces schimperi*	Lyophyllaceae	Dried leaves	Maceration extraction	Methanol	DPPH	33.97 *μ*g/mL	Significant	[27]
*Hibiscus sabdariffa*	Malvaceae	Dried seeds, calyces	Maceration technique	Methanol	DPPH	430 and 140 *μ*g/mL	Low	[36]
*Maesa lanceolata*	Myrsinaceae	Dried leaves	Maceration	Methanol	DPPH	76.7 *μ*g/mL	Moderate	[45]
*Syzygium aromaticum*	Myrtaceae	Dried flowers	Maceration extraction	Methanol	DPPH	303.56 *μ*g/mL	Low	[46]
*Phytolacca dodecandra*	Phytolaccaceae	Dried roots	Maceration extraction	Methanol	DPPH	7.4 *μ*g/mL	Significant	[47]
*Piper capense*	Piperaceae	Dried seeds	Maceration technique	Methanol	DPPH	71.9 *μ*g/mL	Moderate	[26]
*Plumbago zeylanica*	Plumbaginaceae	Dried leaves	Maceration extraction	Methanol	DPPH	53.14 *μ*g/mL	Moderate	[48]
*Rumex nepalensis*	Polygonaceae	Dried roots	Maceration	Ethanol	DPPH	5.7 *μ*g/mL	Significant	[49]
*Cheilanthes farinosa*	Pteridaceae	Dried aerial parts	Soxhlet	Methanol	DPPH	52.5 *μ*g/mL	Moderate	[38]
*Clematis hirsuta*	Ranunculaceae	Dried roots	Maceration	Methanol	DPPH	590 *μ*g/mL	Low	[50]
*Clematis simensis*	Ranunculaceae	Dried stem bark	Maceration extraction	Ethanol	DPPH	42.35 mg/mL	Significant	[51]
*Nigella sativa*	Ranunculaceae	Dried seeds	Maceration technique	Methanol	DPPH	94.1 *μ*g/mL	Moderate	[26]
*Ziziphus spina-christi*	Rhamnaceae	Dried fruits	Soxhlet	Methanol	ABTS	15480 *μ*g/ml	Low	[52]
*Hagenia abyssinica*	Rosaceae	Dried leaves	Maceration extraction	Methanol	DPPH	10.25 *μ*g/mL	Significant	[53]
*Rubus steudneri*	Rosaceae	Dried roots	Maceration	Ethanol	DPPH	5.8 *μ*g/mL	Significant	[49]
*Verbascum sinaiticum*	Scrophulariaceae	Dried leaves	Maceration extraction	Methanol	DPPH	1.70 *μ*g/mL	Significant	[54]
*Datura stramonium*	Solanaceae	Dried roots, seeds	Maceration	Hydro methanol	DPPH	13.47 and 11.95 *μ*g/mL	Significant	[55, 56]
*Gnidia involucrata*	Thymelaeaceae	Dried root barks	Maceration extraction	EtOAc, methanol	DPPH	7.9 and 17.7 *μ*g/mL	Significant	[57]
*Urtica simensis*	Urticaceae	Dried leaves	Maceration extraction	Methanol	DPPH	165.89 *μ*g/mL	Low	[23]
*Lippia adoensis*	Verbenaceae	Dried leaves	Maceration technique	Methanol	DPPH	49.2 *μ*g/mL	Significant	[26]
*Curcuma domestica*	Zingiberaceae	Dried leaves	Maceration extraction	Methanol	DPPH	96.98 *μ*g/mL	Moderate	[27]
		Dried rhizome	Hydrodistillation	Oil	DPPH	23.05 *μ*g/mL	Significant	[58]

**Table 2:** Antioxidant compounds isolated from Ethiopian flora.

Compounds	Plant species	Family	Plant part used	Solvent used	Isolation and identification Method	Assay method	IC_50_ (*μ*g/mL)	Antioxidant potential	Ref
*Flavonoid*									
7, 2′-Dihydroxy-4′-methoxy-6-(3″, 3″-dimethylallyl) isoflavan (**1**)	*Rhynchosia ferruginea*	Fabaceae	Roots	CH_2_Cl_2_/CH_3_OH	TLC, CC, NMR	DPPH	32	Low	[41]
7-Hydroxy-2′, 4′ di-methoxy-8-(2‴, 3‴-dihydroxy-3‴-methylbutyl)-5′- (3″, 3″-dimethylallyl) isoflav-3-ene (**2**)	*Rhynchosia ferruginea*	Fabaceae	Roots	CH_2_Cl_2_/CH_3_OH	TLC, CC, NMR	DPPH	64.5	Low	[41]
Robustaflavone (**3**)	*Rhus ruspolii*	Anacardiaceae	Roots	CH_2_Cl_2_/MeOH	TLC, CC,NMR	DPPH	7.90	Significant	[67]
3-(1-(2,4-Dihydroxyphenyl)-3,3-bis(4-hydroxyphenyl)-1-oxopropan-2-yl)-7-methoxy-4H-chromone-4-one (**4**)	*Rhus ruspolii*	Anacardiaceae	Roots	CH_2_Cl_2_/MeOH	TLC, CC,NMR	DPPH	8.40	Significant	[67]
2′,4′,4″,2‴-Tetrahydroxy-4‴-methoxy-4-*O*-5‴-bichalcone (**5**)	*Rhus ruspolii*	Anacardiaceae	Roots	CH_2_Cl_2_/MeOH	TLC, CC,NMR	DPPH	10.8	Moderate	[67]
Rhuschalcone I (**6**)	*Rhus ruspolii*	Anacardiaceae	Roots	CH_2_Cl_2_/MeOH	TLC, CC,NMR	DPPH	26.03	Low	[67]
Rutin (**7**)	*Cineraria abyssinica*	Asteraceae	Leaves	Aqueous and methanol	TLC, PTLC, NMR	DPPH	3.53	Significant	[31]
	*Cheilanthes farinosa*	Pteridaceae	Aerial parts	Methanol	TLC, CC, NMR	DPPH	5.79	Significant	[38]
	*Euclea racemosa*	Ebenaceae	Leaves	Acetone	TLC, CC, NMR	DPPH	5.79	Significant	[38]
Flavan-3-ol-7-*O*-glucoside (**8**)	*Hydnora johannis*	Hydnoraceae	Roots	CH_2_Cl_2_/MeOH (1 : 1)	TLC, CC, NMR	DPPH	0.190	Significant	[68]
Hyperoside (**9**)	*Bersama abyssinica*	Francoaceae	Leaves	Methanol	TLC, CC, NMR	DPPH	10.49	Moderate	[38]
Quercetin-3-*O*-arabinopyranoside (**10**)	*Bersama abyssinica*	Francoaceae	Leaves	Methanol	TLC, CC, NMR	DPPH	8.99	Significant	[38]
Quercetin-3-*O*-diglucosylrhamnoside (**11**)	*Cheilanthes farinosa*	Pteridaceae	Aerial parts	Methanol	TLC, CC, NMR	DPPH	11.59	Moderate	[38]
Quercetrin (**12**)	*Euclea racemosa*	Ebenaceae	Leaves	Acetone	TLC, CC, NMR	DPPH	12.33	Moderate	[38]
Myricitrin (**13**)	*Euclea racemosa*	Ebenaceae	Leaves	Acetone	TLC, CC, NMR	DPPH	6.59	Significant	[38]
Myricetin-3-*O*-arabinopyranoside (14)	*Euclea racemosa*	Ebenaceae	Leaves	Acetone	TLC, CC, NMR	DPPH	6.99	Significant	[38]
7-*O*-Methylaloeresin A (**15**)	*Aloe harlana*	Asphodelaceae	Leaves' latex	—	TLC, CC, PTLC, NMR	DPPH	0.014	Significant	[29]
*Terpenoids*									
*β*-Stigmasterol (**16**),	*Laggera tomentosa*	Asteraceae	Roots	Methanol	TLC, CC, NMR	DPPH	1150	Low	[33]
3-Hydroxyisoagatholactone (**17**)	*Cyphostemma cyphopetalum*	Vitaceae	Roots	CH_2_Cl_2_/MeOH	TLC, CC, NMR	DPPH	6.05	Significant	[69]
*β*-Sitosterol (**18**)	*Cyphostemma cyphopetalum*	Vitaceae	Roots	CH_2_Cl_2_/MeOH	TLC, CC, NMR	DPPH	2.72	Significant	[69]
	*Hydnora johannis*	Hydnoraceae	Roots	CH_2_Cl_2_/MeOH (1 : 1)	TLC, CC, NMR	DPPH	14.668	Moderate	[68]
Cucurbitacin (**19**)	*Cucumis prophetarum*	Cucurbitaceae	Roots	Methanol	TLC, CC, NMR	DPPH	80.2	Low	[37]
*α*-Spinasterol (**20**)	*Cucumis prophetarum*	Cucurbitaceae	Roots	*n*-Hexane	TLC, CC, NMR	DPPH	172.7	Low	[37]
Spinasterol (**21**)	*Calotropis procera*	Apocynaceae	Roots	CH_2_Cl_2_/MeOH (1 : 1)	TLC, CC, NMR	DPPH	0.3	Significant	[24]
*β*-Sitosterol-3-*O*-*β*-D-glucoside (**22**)	*Hydnora johannis*	Hydnoraceae	Roots	CH_2_Cl_2_/MeOH	TLC, CC, NMR	DPPH	0.014	Significant	[68]

*Anthraquinone*									
Aloin (**23**)	*Aloe harlana*	Asphodelaceae	Leaves' latex	—	TLC, CC, PTLC, NMR	DPPH	41.84	Low	[29]
Microdontin A/B (**24**)	*Aloe schelpei*	Asphodelaceae	Leaves' latex	—	PTLC, NMR	DPPH	0.07	Significant	[30]
Aloin A/B (**25**)	*Aloe schelpei*	Asphodelaceae	Leaves' latex	—	PTLC, NMR	DPPH	0.15	Significant	[30]
Aloinoside A/B (**26**)	*Aloe schelpei*	Asphodelaceae	Leaves' latex	—	PTLC, NMR	DPPH	0.13	Significant	[30]
Chrysophanol (**27**)	*Laggera tomentosa*	Asteraceae	Roots	Methanol	TLC, CC, NMR	DPPH	6.2	Significant	[33]
Emodin (**28**)	*Laggera tomentosa*	Asteraceae	Roots	Methanol	TLC, CC, NMR	DPPH	3.8	Significant	[33]

*Stilbenoids*									
*ε*-Viniferin (**29**)	*Cyphostemma cyphopetalum*	Vitaceae	Roots	CH_2_Cl_2_/MeOH	TLC, CC, NMR	DPPH	0.017	Significant	[69]
Trans-Resveratrol (**30**)	*Cyphostemma cyphopetalum*	Vitaceae	Roots	CH_2_Cl_2_/MeOH	TLC, CC, NMR	DPPH	0.052	Significant	[69]
Gnetin H (**31**)	*Cyphostemma cyphopetalum*	Vitaceae	Roots	CH_2_Cl_2_/MeOH	TLC, CC, NMR	DPPH	0.063	Significant	[69]
*ε*-Viniferin Diol (**32**)	*Cyphostemma cyphopetalum*	Vitaceae	Roots	CH_2_Cl_2_/MeOH	TLC, CC, NMR	DPPH	0.157	Significant	[69]
Parthenostilbenin B (**33**)	*Cyphostemma cyphopetalum*	Vitaceae	Roots	CH_2_Cl_2_/MeOH	TLC, CC, NMR	DPPH	0.025	Significant	[69]

*Alkaloids*									
13-*O*-Pyrrolecarboxyl lupanine (**34**)	*Cadia purpurea*	Fabaceae	Roots	MeOH	TLC, CC, NMR	DPPH	58.44	Low	[44]
*Organic acid*									
Tetratriacontanyl caffeate (**35**)	*Gnidia involucrata*	Thymelaeoideae	Root barks	EtOAC	TLC, CC, NMR	DPPH	73	Low	[57]
12-*O*-Dodeca-2,4-dienoylphorbol-13-acetate (**36**)	*Gnidia involucrata*	Thymelaeoideae	Root barks	EtOAC	TLC, CC, NMR	DPPH	84.9	Low	[57]
(E)-Octadec-7-enoic acid (**37**)	*Crinum abyssinicum*	Amaryllidaceae	Roots	CH_2_Cl_2_/MeOH (1 : 1)	TLC, CC, NMR	DPPH	10.1	Moderate	[24]
Myristic acid (**38**)	*Cucumis prophetarum*	Cucurbitaceae	Roots	*n*-Hexane	TLC, CC, NMR	DPPH	232.3	Low	[37]
Caffeic acid (**39**)	*Cheilanthes farinosa*	Pteridaceae	Aerial parts	Methanol	TLC, CC, NMR	DPPH	4.19	Significant	[38]
Chlorogenic acid (**40**)	*Cheilanthes farinosa*	Pteridaceae	Aerial parts	Methanol	TLC, CC, NMR	DPPH	8.01	Significant	[38]
1, 3-Dilinoleoyl-2-stearoylglycerol (**41**)	*Rhynchosia ferruginea*	Fabaceae	Roots	CH_2_Cl_2_/CH_3_OH	TLC, CC, NMR	DPPH	90.6	Low	[41]

*Xanthonoid*									
Mangiferin (**42**)	*Bersama abyssinica*	Francoaceae	Leaves	Methanol	TLC, CC, NMR	DPPH	6.72	Significant	[38]

*Miscellaneous*									
Di-(2-methylheptyl) phthalate (**43**)	*Cadia purpurea*	Fabaceae	Roots	MeOH	TLC, CC, NMR	DPPH	7.99	Significant	[44]
Ethyl (E)-octadec-8-enoate (**44**)	*Crinum abyssinicum*	Amaryllidaceae	Roots	CH_2_Cl_2_/MeOH (1 : 1)	TLC, CC, NMR	DPPH	3.3	Significant	[24]
(4Z)-Dodec-4-en-1-ol (**45**)	*Calotropis procera*	Apocynaceae	Roots	CH_2_Cl_2_/MeOH (1 : 1)	TLC, CC, NMR	DPPH	7.9	Significant	[24]
Penicilloitins B (**46**)	*Crinum abyssinicum*	Amaryllidaceae	Roots	CH_2_Cl_2_/MeOH (1 : 1)	TLC, CC, NMR	DPPH	8.4	Significant	[24]

## Data Availability

The data used in this study are included within the article.
